# Lack of the Bacterial Phytochrome Protein Decreases *Deinococcus radiodurans* Resistance to Mitomycin C

**DOI:** 10.3389/fmicb.2021.659233

**Published:** 2021-07-30

**Authors:** Jong-Hyun Jung, Soyoung Jeong, Seonghun Im, Min-Kyu Kim, Ho Seong Seo, Sangyong Lim

**Affiliations:** ^1^Radiation Research Division, Korea Atomic Energy Research Institute, Jeongeup, South Korea; ^2^Department of Radiation Science and Technology, University of Science and Technology, Daejeon, South Korea; ^3^Department of Food and Animal Biotechnology, Research Institute of Agriculture and Life Sciences, Seoul National University, Seoul, South Korea

**Keywords:** *Deinococcus radiodurans*, bacteriophytochrome, BphP, BphR, light illumination, mitomycin C resistance

## Abstract

*Deinococcus radiodurans* known for its extraordinary resistance to ionizing radiation contains bacterial phytochrome (BphP), a member of the family of red/far-red light-sensing proteins. In this study, we constructed a *bphP* mutant strain (Δ*bphP*) to investigate the role of *D. radiodurans* BphP (DrBphP) in the DNA damage response. When cells were incubated under light and dark conditions following exposure to DNA damaging agents, such as γ- and UV-radiation and mitomycin C (MMC), no significant difference in cell survival was observed between the wild-type *D. radiodurans* strain (WT) and Δ*bphP*. However, when continuously exposed to MMC under light conditions, the WT strain notably exhibited increased survival compared to cells grown in the dark. The increased survival was not observed in the Δ*bphP* strain. These results are indicative of the protective role of light-activated DrBphP in the presence of MMC. Site-directed mutagenesis revealed that the conserved amino acids Cys-24 and His-532 involved in chromophore binding and signal transduction, respectively, were essential for the protective function of DrBphP. Inactivation of the cognate response regulator (RR; DrBphR) of DrBphP increased MMC resistance in the dark. *In trans* complementation of the *bphP bphR* double mutant strain (Δ*bphPR*) with DrBphR decreased MMC resistance. Considering that DrBphP acts as a light-activated phosphatase that dephosphorylates DrBphR, it appears that phosphorylated DrBphR exerts a negative effect on cell survival in the presence of MMC. DrBphP overexpression resulted in an increase in MMC resistance of Δ*bphPR*, implying that other RRs might be involved in the DrBphP-mediated signaling pathway. A mutant lacking the *dr*_*0781* gene (Δ*dr*_*0781*) demonstrated the same MMC phenotype as Δ*bphR*. Survival was further increased in the *bphR dr*_*0781* double mutant strain compared to each single mutant Δ*bphR* or Δ*dr*_*0781*, suggesting that DR_0781 is also involved in the DrBphP-dependent MMC sensitivity. This study uncovered a previously unknown phenomenon of red/far-red light-dependent DNA damage survival mediated by BphP by identifying the conditions under which DrBphP exhibits a fitness advantage.

## Introduction

*Deinococcus radiodurans*, a small, red-pigmented, tetrad-forming Gram-positive bacterium, is noted for its extreme resistance to a variety of DNA-damaging agents, including γ-radiation, UV radiation, desiccation, mitomycin C (MMC), and hydrogen peroxide (H_2_O_2_; Cox and Battista, 2005). The extreme resistance of *D. radiodurans* has been attributed to its highly efficient DNA repair systems (Cox and Battista, 2005). *Deinococcus radiodurans* possesses an extended synthesis-dependent strand annealing (ESDSA) system, in which RecFOR proteins play essential roles in DNA double-strand break repair (Bentchikou et al., 2010). Plentiful antioxidant systems, including common reactive oxygen species (ROS) scavenging enzymes (e.g., catalase and superoxide dismutase) and a variety of non-enzymatic antioxidants (e.g., carotenoids and manganese species), also contribute to the protection of *D. radiodurans* from DNA-damaging agents (Qi et al., 2020). Since the genome of *D. radiodurans* was sequenced in 1999 (White et al., 1999), proteins involved in both the DNA repair and antioxidant systems have been investigated (Makarova et al., 2001; Slade and Radman, 2011; Lim et al., 2019).

**Figure 1:**
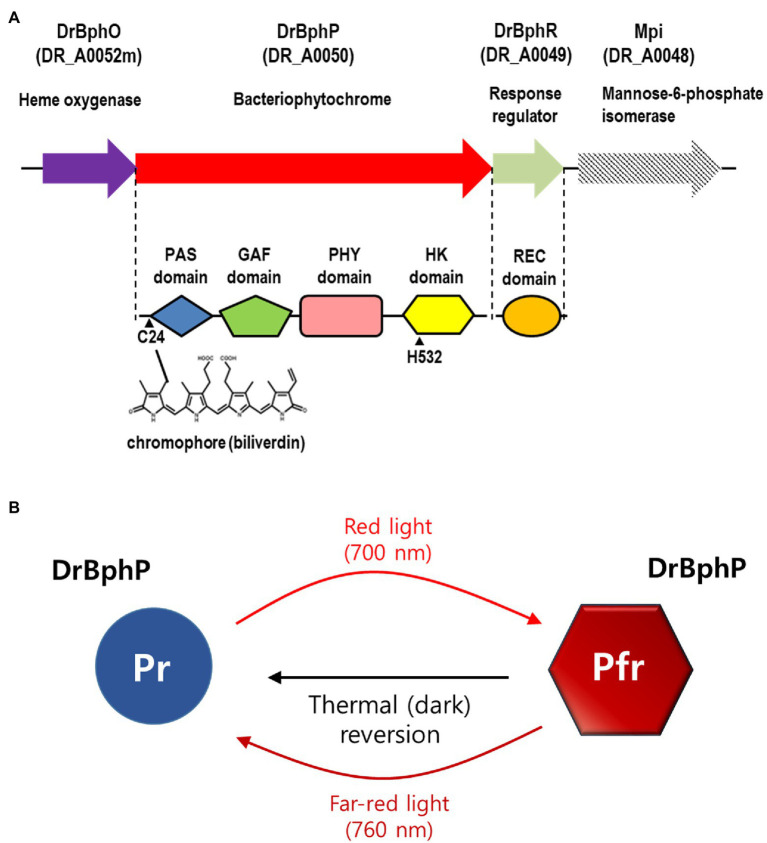
Domain architecture and photocycle of *Deinococcus radiodurans* BphP (DrBphP). **(A)** Schematic representation of the *D. radiodurans bph* operon. Top line represents the *bph* operon and the downstream gene *mpi*. Each protein is shown with its corresponding locus tag. The Period/Arnt/Single-Minded (PAS), cGMP phosphodiesterase/adenylyl cyclase/FhlA (GAF), phytochrome region (PHY), and histidine kinase (HK) domains are linearly arranged in DrBphP. C24 that covalently binds biliverdin and H532 that interacts with DrBphR are indicated. **(B)** DrBphP photoconversion. Pr and Pfr denote the red and far-red light-absorbing conformations of DrBphP, respectively, which are reversible depending on light conditions. Pfr can also be converted to Pr in a light-independent process, referred to as thermal or dark reversion.

Phytochromes are red/far-red reversible photoreceptors that regulate a wide array of light-dependent processes in plants and microorganisms (Burgie and Vierstra, 2014). Plant phytochromes act as light-regulated master switches in various photomorphogenic processes, such as seed germination, de-etiolation, stem growth, pigmentation, and flowering (Vierstra and Zhang, 2011). The bacterial phytochrome, BphP, from *D. radiodurans* was the first BphP to be discovered in non-photosynthetic bacteria and was evaluated and described in detail in 1999 (Davis et al., 1999). Classical phytochromes are homodimeric complexes with each subunit containing an N-terminal photosensory module (PSM) that covalently binds a bilin chromophore and a C-terminal module, often a histidine kinase (HK), responding to the light signal (Vierstra and Zhang, 2011; Burgie and Vierstra, 2014). The most common PSM architecture involves three domains, Period/Arnt/Single-Minded (PAS), cGMP phosphodiesterase/adenylyl cyclase/FhlA (GAF), and phytochrome region (PHY; Vierstra and Zhang, 2011; Burgie and Vierstra, 2014). *Deinococcus radiodurans* BphP (DrBphP) contains the PSM (PAS-GAF-PHY) linked to the C-terminal HK module (Figure 1A; Burgie and Vierstra, 2014).

Phytochromes exist in two states that absorb red light (Pr state) and far-red light (Pfr state; Takala et al., 2015). DrBphP undergoes rapid inter-conversion between the Pr and Pfr states upon absorption of the appropriate wavelength as follows: Pr is converted to Pfr following red light (700 nm) exposure, and Pfr is converted to Pr by far-red light (760 nm; Figure 1B; Bhoo et al., 2001). Based on the observation that red light increased pigmentation in *D. radiodurans*, but far-red light did not, it was suggested that DrBphP is more active in its Pfr form (Davis et al., 1999; Bhoo et al., 2001). Also, it has been reported that DrBphP helps to protect the bacterium from visible light: colony growth of DrBphP-deficient strain was reduced compared to wild type when grown under intense light conditions (Davis et al., 1999). Many BphPs containing HK domains function as typical sensor kinases of two-component signaling systems (TCSs). Following autophosphorylation, a HK transfers the phosphoryl group to a response regulator (RR), which then regulates gene expression or other processes, thus facilitating adaption to environmental changes (Laub and Goulian, 2007). In *D. radiodurans*, the *bphP* gene immediately precedes the *bphR* gene, which encodes a putative cognate RR of DrBphP, named DrBphR (Figure 1A). However, a recent study showed that DrBphP lacks autokinase or phosphotransfer activities in both the Pr and Pfr states; thus it does not produce phosphorylated DrBphR (phospho-DrBphR; Multamäki et al., 2020). Instead, DrBphP functions exclusively as a light-activated phosphatase. DrBphP can dephosphorylate phospho-DrBphR, which is phosphorylated using a non-enzymatic mechanism or by other HKs (Multamäki et al., 2020).

Despite intensive physicochemical and biochemical analysis of DrBphP, the DrBphP functional roles in DNA damage resistance have not been investigated. In this study, we constructed a *bphP* mutant strain to investigate whether DrBphP contributes to the *D. radiodurans* resistance to DNA-damaging agents. Our results revealed that light-activated DrBphP contributes to bacterial resistance to genotoxic stress caused by MMC, an alkylating agent that induces DNA inter-strand cross-links (ICLs) inhibiting DNA synthesis.

## Materials and Methods

### Bacterial Strains and Growth Conditions

*Deinococcus radiodurans* R1 (ATCC 13939) was obtained from the American Type Culture Collection (ATCC). *Deinococcus wulumuqiensis* (KACC 16755; DSM 28115), *Deinococcus maricopensis* (KACC 11804; DSM 21211), and *Deinococcus proteolyticus* (KACC 12249; ATCC 35074) were obtained from the Korea Agricultural Culture Collection (KACC). *Deinococcus* cells were grown at 30°C in TGY (0.5% tryptone, 0.3% yeast extract, and 0.1% glucose) broth or on TGY plates supplemented with 1.5% Bacto-agar. *Escherichia coli* DH5α was used for routine cloning experiments. *Escherichia coli* strains were grown at 37°C in Luria-Bertani (LB) medium or on LB plates solidified with agar. Transformed *E. coli* cells were selected using ampicillin (100 μg/ml) or kanamycin (50 μg/ml), as necessary.

### Construction of Mutant Strains

A mutant strain lacking *bphP* was constructed using fusion PCR, as previously described (Im et al., 2013a). The primers used are listed in Supplementary Table S1. Each up and downstream region of *bphP* was amplified using the upstream (bphP-Up) and downstream (bphP-Dn) primer pairs, respectively. These two PCR fragments were mixed and amplified by PCR using their outer primers, i.e., the bphP-Up forward and bphP-Dn reverse primers, to produce a single amplicon. The fusion PCR product was then cloned into a pGEM-T easy vector (Promega, Madison, WI, United States), digested with NruI, and ligated to a 975 bp HincII fragment (*kan* cassette) from pKatAPH3 (Im et al., 2013a). The mutant strains lacking *bphP* were constructed by transforming wild-type *D. radiodurans* strain with the PCR products amplified from the recombinant plasmid. A *bphR* mutant strain was constructed as described above using the bphR-Up and bphR-Dn primer sets. To construct a *bphPR* double mutant strain, the DNA fragment containing *bphP* and *bphR* was amplified using bphPR forward and reverse primers and then cloned into the pGEM-T easy vector. Because the *bphR* and *bphP* genes have internal SmaI and StuI sites, respectively, the recombinant pGEM-T plasmid was ligated to the *kan* cassette from pKatAPH3 following enzyme digestion. Other *D. radiodurans* RR mutant strains were constructed using the deletion mutagenesis technique, as previously described (Im et al., 2013b). Briefly, the up and downstream regions (approximately 1 kb) of each of the target genes were amplified using the appropriate primer pairs (Supplementary Table S1). The amplified PCR fragments were digested with XhoI (or KpnI)/EcoRV to produce the upstream fragments and XbaI/PstI to produce the downstream fragments, respectively, and then cloned into the corresponding sites of pKatAPH3. The final selection of the transformed *D. radiodurans* cells was completed using kanamycin (8 μg/ml) supplementation. For the construction of *dr*_*0781 bphR* and *dr*_*0781 bphR*, double mutant strains pKatCAT5 with a chloramphenicol-resistance gene cassette was used (Satoh et al., 2009). The up and downstream regions of *dr*_*0781* were amplified using the primer pairs dr0781-Up-Cam and dr0781-Dn-Cam, respectively (Supplementary Table S1). The PCR-amplified up and downstream regions were digested with XhoI/EcoRV and BamHI/PstI, respectively, and then cloned into the corresponding sites of pKatCAT5. The resulting plasmid was transformed into the *bphR* or *bphP* mutant strain, and the cells were incubated on TGY plates supplemented with chloramphenicol (3.8 μg/ml). Gene replacement was confirmed by PCR and nucleotide sequencing.

### Plasmid Construction

The *bphP* and *bphR* gene expression plasmids pBP and pBR were constructed as previously described (Jeong et al., 2016). The pRADgro plasmid, which contains the *groESL* promoter for constitutive gene expression, was used as a backbone plasmid (Jeong et al., 2016). The forward and reverse primers for *bphP* and *bphR* amplification were designed to include ApaI and HindIII sites, respectively, to facilitate the ligation of the PCR products (Supplementary Table S1). Single amino acid substitutions were introduced into DrBphP using complementary primer pairs, BphP-C24A and BphP-H532A, which contain the C24A and H53A mutations, respectively (Supplementary Table S1). Site-directed mutagenesis was carried out under the following conditions: 50 ng of pBphP plasmid, 1 μM of each primer, 0.2 mM of each dNTP, 1 U of nPfu-Forte polymerase, and 10× nPfu-Forte buffer (Enzynomics, Daejeon, Korea) were mixed in a final volume of 50 μl and then amplified using the following cycling parameters: cycling protocols were 2 min at 95°C followed by 18 cycles of 30 s at 95°C, 30 s at 55°C, 7 min at 68°C, and a final extension of 10 min at 72°C. The amplified plasmids, which were designated as pBP_C_ and pBP_H_, respectively, were digested with DpnI and transformed into *E. coli* DH5α. Nucleotide sequences of the target genes inserted into pRADgro were verified by sequencing. The transformed *D. radiodurans* cells were selected using chloramphenicol (3.4 μg/ml) supplementation.

### Acute Exposure Assays

*Deinococcus radiodurans* cultures grown to log phase (OD_600_ ≈ 1.0) were adjusted to ~10^7^ CFU/ml in TGY medium and exposed to different DNA-damaging agents. Cells were treated as described below, serially diluted 10-fold in 0.85% NaCl, and spotted on TGY plates. The plates were then incubated in the dark or under red light (700 nm) and far-red light (760 nm) for 2 days (LED light incubator DS210-SF, Daewon Science, Korea). Dimensions of an experimental chamber were 500 mm wide × 500 mm high × 500 mm deep, with a surface area of 0.250 m^2^. The chamber contained 16 LED strips, eight for each light, 700 nm and 760 nm, in its ceiling, and samples were placed at a distance of 10 mm from the ceiling. Each LED strip was manufactured with 36 LEDs (5,050 SMD LED, 0.3 lumen). Resistance to MMC or H_2_O_2_ was determined by adding different concentrations of MMC or H_2_O_2_ to *D. radiodurans* cultures grown in TGY. The cultures were incubated for 1 h in the presence of MMC or H_2_O_2_ with shaking at 30°C and then evaluated for viability. The γ-radiation treatment was completed by irradiating the bacterial cells at room temperature using a ^60^Co-gamma irradiator (AECL, IR-79; MDS Nordion International Co. Ltd., Ottawa, Canada) at the Advanced Radiation Technology Institute in the Republic of Korea. The dose rate was 6 kGy/h. UV-stress survival was evaluated by exposing plates, on which cells were spotted, to UVC light in a UVC ultraviolet crosslinker (CX-2000, UVP LLC, Upland, CA, United States) at 20 J/m^2^/s for different time intervals.

### Chronic Exposure Assays

The cells were grown to log phase (OD_600_ ≈ 1.0), adjusted to ~10^7^ CFU/ml, and then serially diluted 10-fold in fresh TGY medium from 10^7^ to 10^2^ CFU/ml. Then, 10-μl (10^5^–10^0^ CFU) of each dilution was spotted on TGY plates supplemented with various concentrations of MMC, H_2_O_2_, methyl methanesulfonate, or toluidine blue, and then incubated for 2 days under different light conditions as described in the “Acute Exposure Assays” section.

### Quantitative Real-Time PCR

A 5 ml culture was grown to the early log phase (OD_600_ ≈ 1.0) under red light and dark conditions and then used in RNA isolation and cDNA synthesis as previously described (Jeong et al., 2016). Quantitative real-time PCR (qRT-PCR) was performed on a CFX Connect real-time PCR system (Bio-Rad) using SYBR Premix Ex Taq (Takara Bio Inc., Otsu, Japan). PCR reactions were performed as follows: one cycle of 95°C for 5 min, followed by 40 cycles of 95°C for 10 s and 60°C for 30 s. The housekeeping gene, *dr1343*, encoding glyceraldehyde-3-phosphate dehydrogenase, was used as an internal control. The primers used in these assays are summarized in Supplementary Table S1.

## Results

### *bphP* Mutation Abolishes Light-Dependent MMC Resistance in *D. radiodurans*

**Figure 2:**
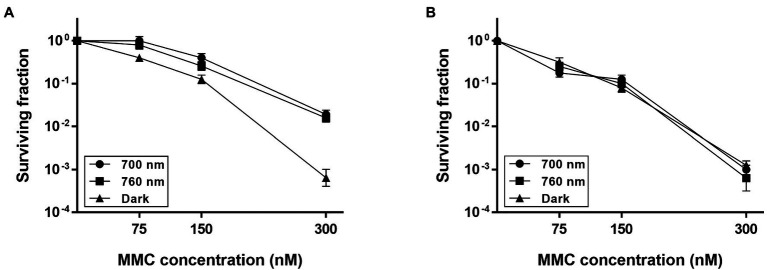
Survival of Δ*bphP* during continuous exposure to mitomycin C (MMC). *Deinococcus radiodurans* wild-type **(A)** and *bphP* mutant **(B)** strains were grown to log phase, then serially diluted, and spotted on TGY plates supplemented with the indicated concentrations of MMC. The plates were incubated under red light (700 nm), far-red light (760 nm), or no light (dark) conditions for 2 days prior to the enumeration of colonies. The surviving fraction was calculated by dividing the CFUs of MMC-exposed cells by the CFUs of non-exposed cells. The error bars represent the SD of three independent experiments (*n* = 3).

To investigate whether DrBphP contributes to cell survival, first, we treated *D. radiodurans* wild-type (WT) and *bphP* mutant (Δ*bphP*) strains with DNA-damaging agents and then incubated the strains under red light (700 nm) and no light (dark) conditions. A 12 kGy dose of γ-radiation resulted in an approximately 1-log reduction in ∆*bphP* survival relative to WT, but this reduction was observed under both light and dark conditions (Supplementary Figure S1). When treated with MMC and UV-C radiation, the survival of WT and Δ*bphP* was dose-dependently reduced, but there was no difference in survival between the two strains (Supplementary Figure S2). Next, we evaluated their abilities to withstand DNA-damaging agents in the light. To this end, cells were dotted onto plates supplemented with MMC and incubated under red or far-red light (760 nm) and in the dark. Compared to the acute exposure method, *D. radiodurans* cells were shown to be highly sensitive to MMC in this chronic assay. WT survival was reduced by approximately 3 log units in the dark when cells were exposed to 300 nM (≈ 100 ng/ml) of MMC (Figure 2A), which was equivalent to the survival rate of WT treated with 30 μM of MMC for 1 h in the acute assay (Supplementary Figure S2). The increased sensitivity is likely attributable to the continuous challenge with MMC over a 2-day incubation period, thus, even relatively low levels of MMC can exert significant detrimental effects on cellular survival.

**Figure 3:**
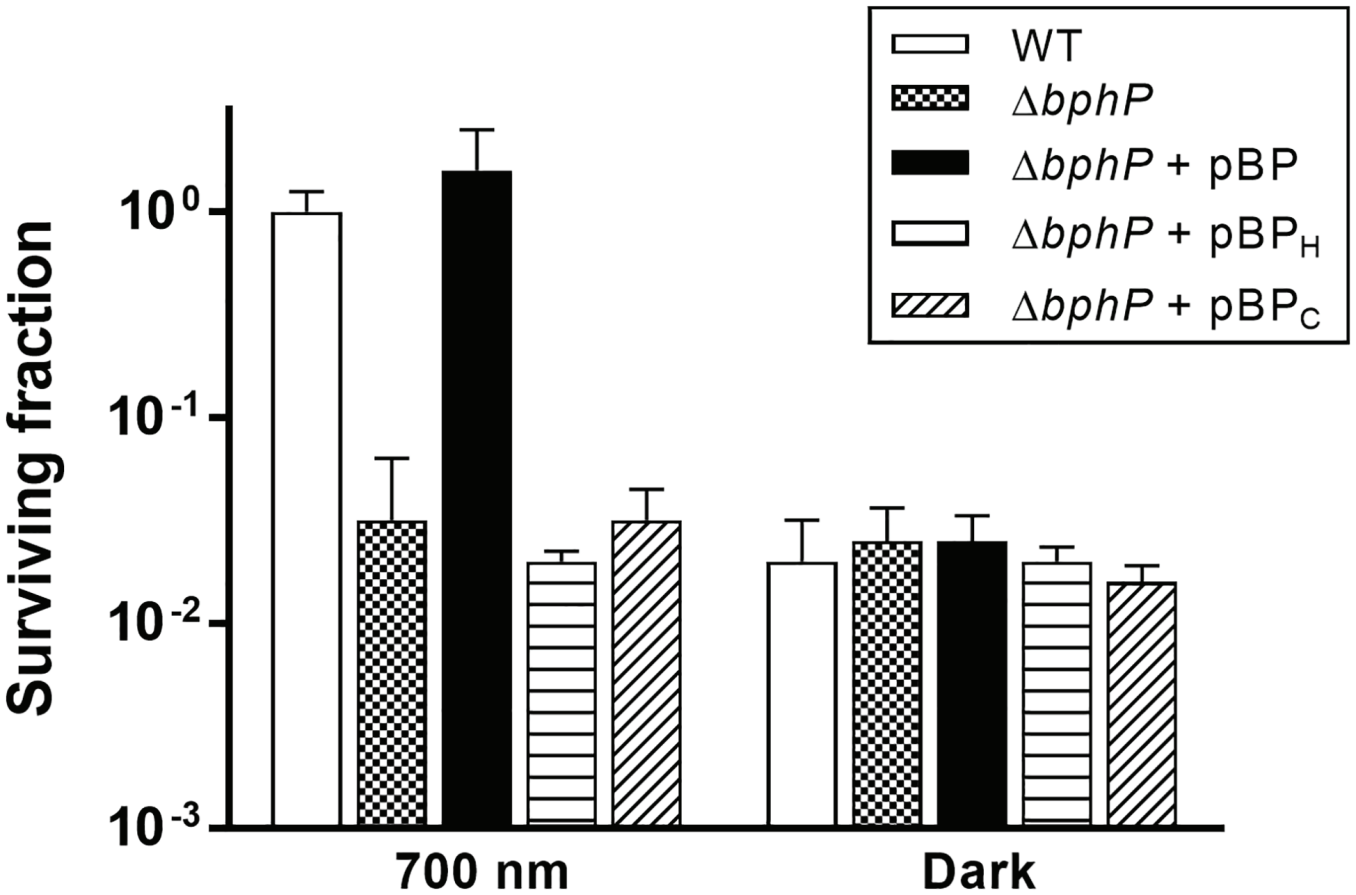
Complementation of Δ*bphP*. *Deinococcus radiodurans* wild-type and *bphP* mutant strains harboring the *bphP* expression plasmids were spotted on TGY plates supplemented with 300 nM MMC. The pBP expresses the wild type DrBphP, and the pBP_H_ and pBP_C_ plasmids encode DrBphP H532A and C24A mutants, respectively. The plates were incubated under red light (700 nm) or no light (dark) conditions for 2 days prior to the enumeration of colonies. The surviving fraction was expressed relative to light-grown wild-type (WT) survival (arbitrarily set at 1). The error bars represent the SD of three independent experiments (*n* = 3).

The WT strain grown in the dark showed a significant reduction in survival at 300 nM MMC when compared to WT grown under light conditions (Figure 2A), while there was no difference in the survival of Δ*bphP* grown under all conditions tested (Figure 2B). Interestingly, the survivability of *∆bphP* was similar to that of WT grown in the dark. When exposed to H_2_O_2_ or methyl methanesulfonate (MMS), another DNA alkylating agent, in the same way described above Δ*bphP* exhibited the same degree of resistance to these agents as the WT (Supplementary Figure S3). We constructed an expression plasmid producing DrBphP and used this construct to complement the Δ*bphP* strain. Complementation by plasmid-borne *bphP in trans* fully restored the growth-defective phenotype of Δ*bphP* (Figure 3). This clearly demonstrates that the decreased MMC resistance in Δ*bphP* can be attributed to the lack of DrBphP. Taken together, our results show that DrBphP is specifically required for resistance to continuous MMC exposure and that photoactivation of DrBphP by illumination is a prerequisite for its protective function.

### Cys-24 and His-532 Are Critical to DrBphP Function

*Deinococcus radiodurans* BphPs use BV as a chromophore, which is synthesized by the oxidative degradation of heme by heme oxygenase (HO; Bhoo et al., 2001). *Deinococcus radiodurans* possesses a *bphO* gene encoding its own HO, which is located upstream of *bphP* (Figure 1A). DrBphP binds BV *via* a conserved cysteine (Cys-24) upstream of the PAS domain (Wagner et al., 2005). The DrBphP histidine residue, His-532, is one of the three central DrBphP residues that interact with DrBphR (Multamäki et al., 2020). We constructed DrBphP derivatives using alanine substitution at amino acids Cys-24 and His-532. When the engineered proteins harboring C24A and H532A were provided *in trans*, both C24A and H532A mutants failed to restore the survival of Δ*bphP* to the WT levels under light conditions (Figure 3). These results suggest that the BV-binding Cys24 and DrBphR interacting His532 are crucial for the protective role of DrBphP against MMC in light.

### Deinoxanthin-Deficient Strain Is Not Sensitive to Continuous MMC Exposure

**Figure 4:**
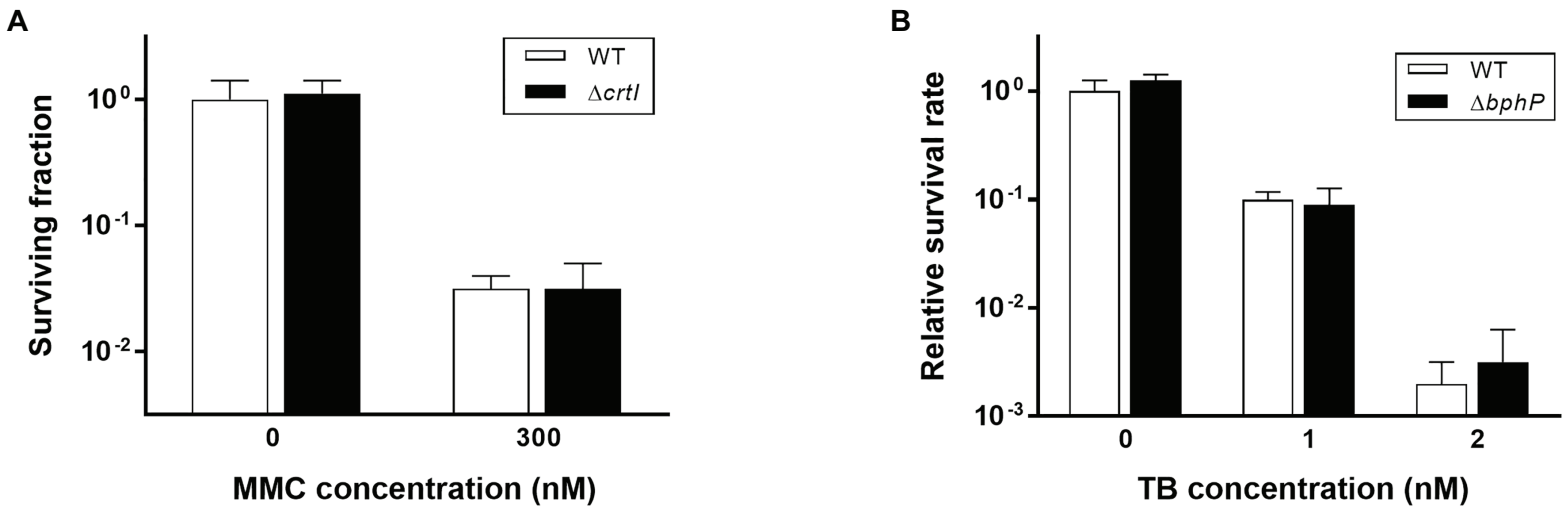
Survival of Δ*crtI* during continuous exposure to MMC **(A)** and survival of Δ*bphP* during continuous exposure to toluidine blue (TB; **B**). The *D. radiodurans* strains were grown to log phase and spotted on TGY plates supplemented with the indicated concentrations of MMC or TB. The plates were incubated under red light (700 nm) conditions for 2 days prior to the enumeration of colonies. The surviving fraction was expressed relative to non-treated WT survival (arbitrarily set at 1). The error bars represent the SD of three independent experiments (*n* = 3).

Mitomycin C is a quinone-containing alkylating agent, and bio-reductive activation of MMC is responsible for its cytotoxic properties. Enzymatic reduction of the quinone moiety in MMC results in the production of various ROS, including superoxide anion and H_2_O_2_ (Wang et al., 2010). It has been reported that DrBphP is involved in the biosynthesis of a unique carotenoid, deinoxanthin (Davis et al., 1999). Because deinoxanthin has ROS-scavenging activity (Tian et al., 2004), we investigated the resistance of the *crtI* mutant strain (*∆crtI*), which lacks the phytoene desaturase necessary for deinoxanthin production (Joe et al., 2012), to determine whether a defect in deinoxanthin could introduce the same phenotype as *∆bphP*. There was no significant difference in MMC resistance between WT and *∆crtI*, suggesting that a change in deinoxanthin availability cannot explain the decreased MMC resistance of *∆bphP* (Figure 4A). Red light induces photodynamic stress by increasing the levels of ROS in the presence of oxygen and photosensitizers (Sperandio et al., 2013). Thus, we examined the growth of WT and *∆bphP* using toluidine blue (TB) as a photosensitizer. Although the survival fractions were reduced in the presence of TB when cells were grown under red light conditions, the difference between the WT and *∆bphP* strains was marginal (Figure 4B), which was consistent with the results of the H_2_O_2_ survival test (Supplementary Figure S3). Taken together, these results imply that ROS neutralization is not directly associated with the MMC-specific protective function of DrBphP.

### *bphR* Mutation Increases MMC Resistance in *D. radiodurans*

**Figure 5:**
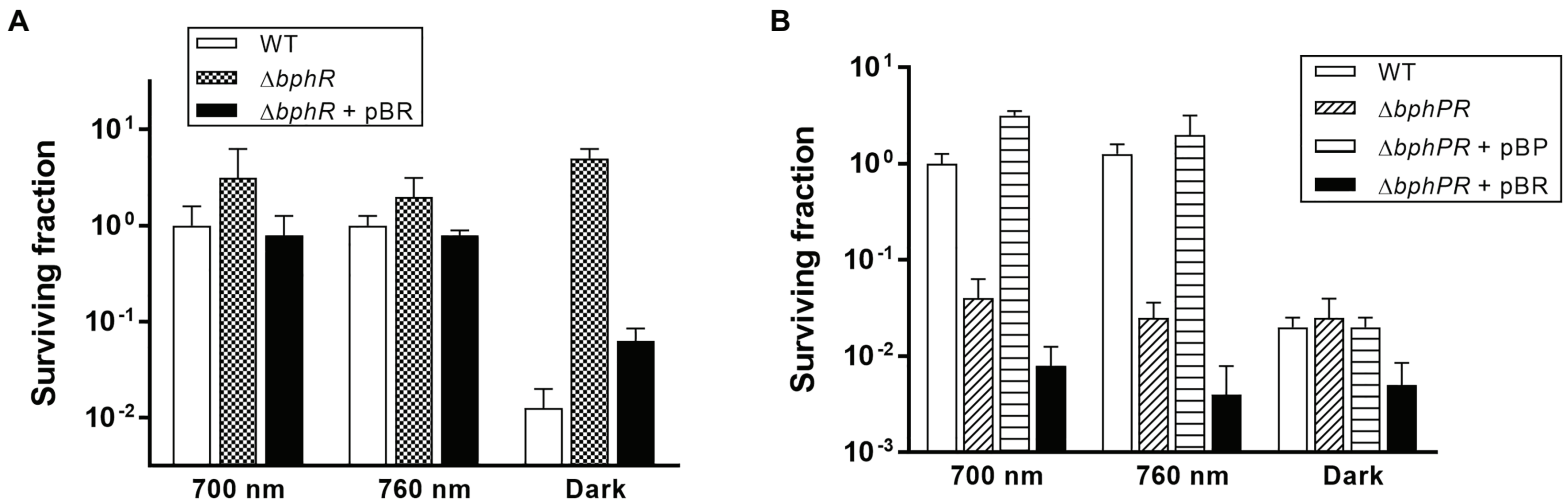
Survival and complementation of Δ*bphR* **(A)** and Δ*bphPR* **(B)**. *Deinococcus radiodurans* wild-type, *bphR*, and *bphPR* mutant strains were grown to log phase, then serially diluted, and spotted on TGY plates supplemented with 300 nM MMC. The plasmids pBR and pBP, which carries the *bphR* and *bphP* genes, respectively, were used for the complementation assay. The plates were incubated under red light (700 nm), far-red light (760 nm), or no light (dark) conditions for 2 days prior to the enumeration of colonies. The surviving fraction was expressed relative to 700 nm-grown WT survival (arbitrarily set at 1). The error bars represent the SD of three independent experiments (*n* = 3).

*Deinococcus radiodurans* BphP dephosphorylates its cognate response regulator (RR), DrBphR (Multamäki et al., 2020). To identify the role of DrBphR in MMC resistance, we constructed a mutant *bphR* (*∆bphR*) strain and subjected this to the same survival assays as described above. The viability of *∆bphR* was similar to that of WT under red and far-red light conditions (Figure 5A). Notably, the surviving fraction of *∆bphR* did not decrease in the dark, which resulted in a clear difference between the ∆*bphR* and WT strains (Figure 5A). The enhanced MMC-resistance phenotype of *∆bphR* was partially complemented by the expression of a plasmid-encoded *bphR* gene, especially in the dark (Figure 5A). The *bphP bphR* double mutant strain (*∆bphPR*) was shown to be sensitive to continuous exposure of MMC, similar to the *∆bphP* strain (Figure 5B). Interestingly, trans-complementation of the *bphP* gene fully restored the MMC resistance of Δ*bphPR* to the wild-type level, while the expression of *bphR* decreased the MMC resistance of Δ*bphPR* (Figure 5B). Considering the opposite effects of the *bphP* and *bphR* mutations, we conclude that DrBphP counteracts the effects of DrBphR in the light, presumably *via* de-phosphorylation.

### DR_0781 Is an Additional RR Affecting MMC Sensitivity

**Figure 6:**
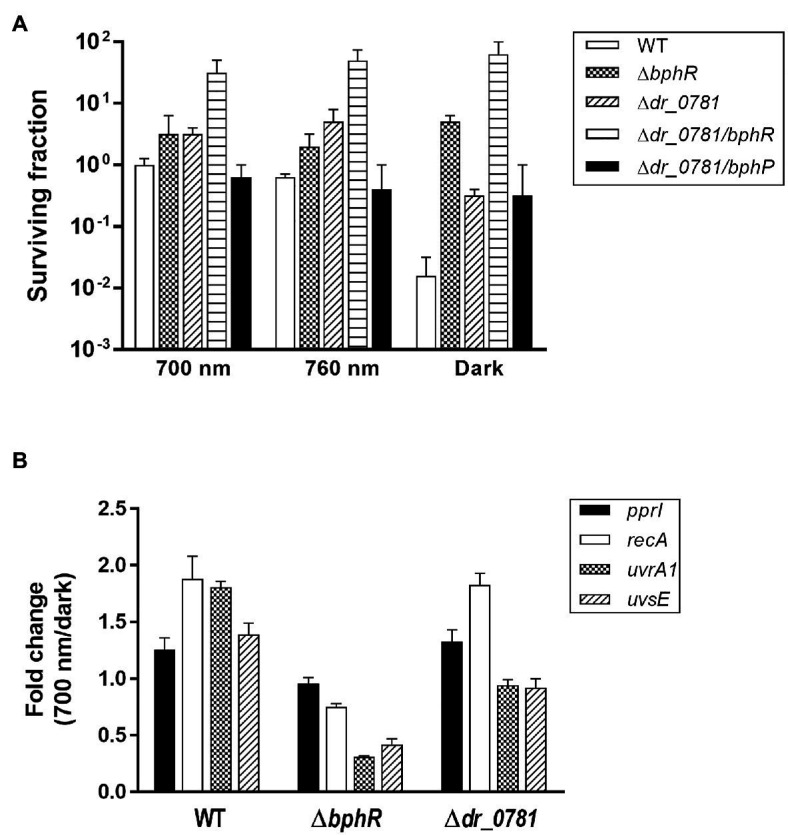
Effect of *dr*_*0781* mutation on MMC resistance. **(A)** *Deinococcus radiodurans* wild-type, *dr*_*0781*, *dr*_*0781 bphR*, and *dr*_*0781 bphP* mutant strains were grown to log phase, then serially diluted, and spotted on TGY plates supplemented with 300 nM MMC. The plates were incubated under red light (700 nm), far-red light (760 nm), or no light (dark) conditions for 2 days prior to the enumeration of colonies. The surviving fraction was expressed relative to 700 nm-grown WT survival (arbitrarily set at 1). The error bars represent the SD of three independent experiments (*n* = 3). **(B)** Quantitative real-time PCR (qRT-PCR) assay. Cells were grown to log phase (OD_600_ ≈ 1.0) in the presence of MMC (300 nM) under red light (700 nm) and dark conditions, respectively. The relative expression values of indicated genes were determined by dividing the mRNA levels from the illuminated cells by the mRNA levels from cells kept in the dark. The expression levels of the target genes were normalized against *dr*_*1343*. The data are represented as the mean ± SD of three independent experiments performed in duplicate.

Photoactivated DrBphP increases MMC resistance in cells lacking DrBphR (Figure 5B), suggesting that there might be other RRs involved in DrBphP-mediated regulation. We found 26 putative RRs in *D. radiodurans*, which possess a RR receiver domain, in the Microbial Signal Transduction database (Gumerov et al., 2020): DR_0408, DR_0432, DR_0731, DR_0743, DR_0781, DR_0891, DR_0987, DR_1175, DR_1418, DR_1558, DR_1605, DR_2245, DR_2556, DR_2327, DR_2415, DR_2418, DR_2420, DR_A0010, DR_A0350, DR_A0357, DR_A0139, DR_A0204, DR_B0028, DR_B0029, DR_B0081, and DR_B0091. We were able to inactivate 11 of these genes, and 12 mutant strains, including the previously constructed *drtR* (*dr*_*2415*) mutant strain (Im et al., 2013b), were evaluated using an MMC survival assay (Supplementary Figure S4). There were no strains whose survival fraction decreased as much as Δ*bphP* under red light conditions, but Δ*dr*_*0781* survival was not reduced in the dark like Δ*bphR* (Supplementary Figure S4). We constructed a *dr*_*0781 bphR* double mutant strain (Δ*dr*_*0781*/*bphR*) and evaluated its ability to withstand MMC. Δ*dr*_*0781*/*bphR* exhibited an enhanced MMC resistance under all conditions tested compared to each single mutant Δ*dr*_*0781* or Δ*bphR* (Figure 6A), suggesting that DR_0781 and DrBphR attenuate MMC resistance *via* independent pathways.

In *D. radiodurans*, repair of MMC-induced ICLs involves multiple DNA repair pathways such as nucleotide excision repair and homologous recombination, in which UvrABC, UvsE, and RecA act cooperatively (Slade and Radman, 2011). *Deinococcus*-specific regulatory protein PprI, also called IrrE, is required for the induced expression of *uvrA* and *recA* (Lim et al., 2019). We compared expression of the *pprI*, *recA*, *uvrA1*, and *uvsE* genes between WT and mutants. Neither gene mutations nor light illumination affected the *pprI* expression significantly (Figure 6B). Interestingly, *recA* expression was induced by light illumination in WT and Δ*dr*_*0781*, but not in Δ*bphR* (Figure 6B). Light also enhanced *uvrA1* expression in WT, but not in Δ*bphR* and Δ*dr*_*0781*, and *uvrA1* and *uvsE* were downregulated in Δ*bphR* (Figure 6B). When the *bphR* mutation was complemented *in trans*, the gene expression pattern was restored to that of WT (Supplementary Figure S5). The different gene expression profiles of Δ*bphR* and Δ*dr*_*0781* are consistent with our conclusions that DrBphR and DR_0781 function independent of each other.

### The Protective Role of BphP Is Observed in Other *Deinococcus* Species

**Figure 7:**
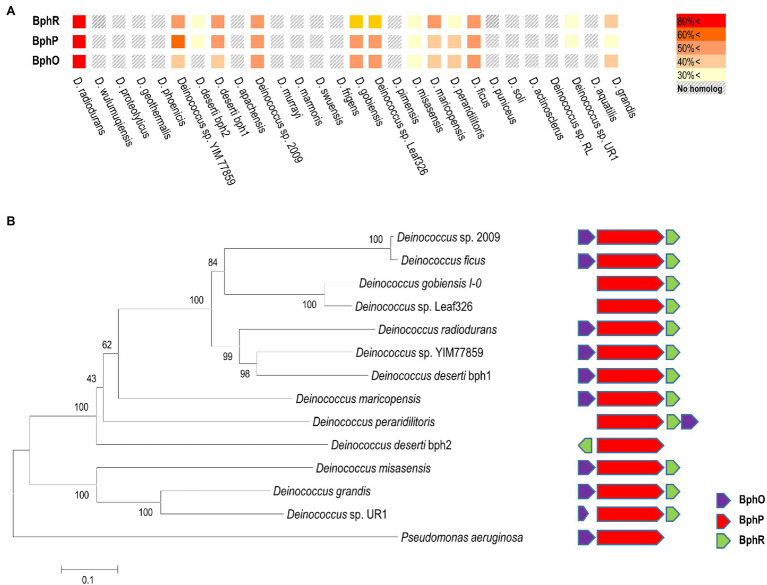
Distribution of the Bph proteins among several *Deinococcus* species. Amino acid sequences of Bph proteins were obtained from the genomes of 27 *Deinococcus* strains in the NCBI database, and sequence homology analysis was performed using BLAST. Colors in the heat map represent homology between Bph proteins, when compared to the *D. radiodurnas* BphR, BphP, and BphO proteins, respectively **(A)**. The phylogenetic analysis was carried out using a protein sequence alignment of 13 deinococcal BphP and *Pseudomonas aeruginosa* BphP proteins completed in Clustal omega. The phylogenetic tree was developed using the neighbor-joining algorithm in MEGA 6.0, and the scale indicates the number of amino acid substitutions per site. All the node numbers represent the bootstrap value based on 1,000 replications (left side panel in **B**). Comparison of the gene order of related *bph* operons (right side panel in **B**).

**Figure 8:**
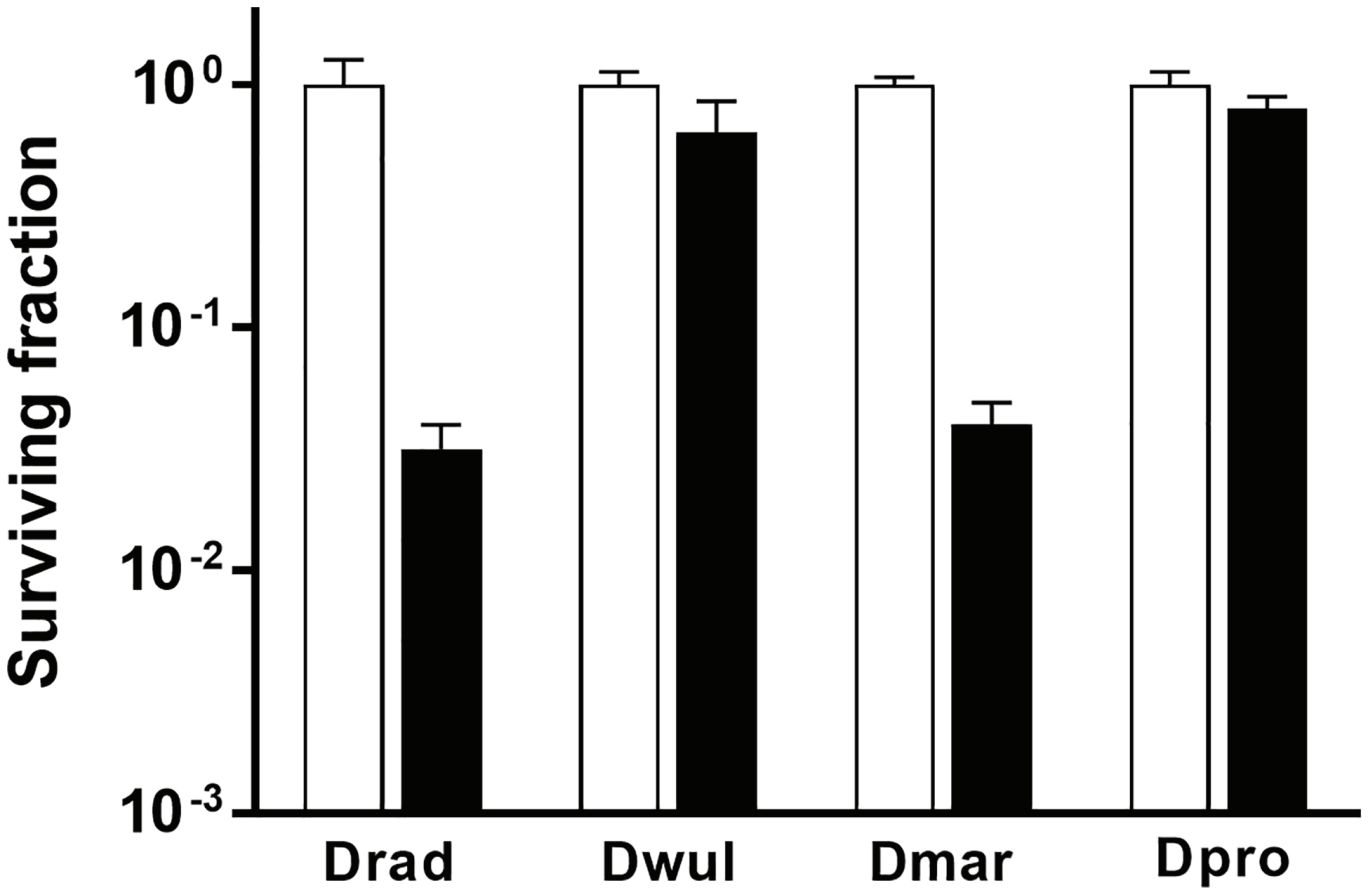
Survival of *Deinococcus* species with or without BphP in response to MMC. The *bphP*-positive strains, *D. radiodurans* (Drad) and *D*. *maricopensis* (Dmar), and the *bphP*-negative strains, *D. wulumuqiensis* (Dwul) and *D*. *proteolyticus* (Dpro), were grown to log phase and spotted on TGY plates supplemented with 300 nM MMC. The plates were incubated under red light (700 nm) or no light (dark) conditions for 2 days prior to the enumeration of colonies. The surviving fraction was calculated by dividing the CFUs of dark-grown cells by the CFUs of light-grown cells. The error bars represent the SD of three independent experiments (*n* = 3).

In *D. radiodurans*, *bphO*, *bphP*, and *bphR* constitute the *bph* operon (Figure 1A). We analyzed the distribution of BphP in 27 *Deinococcus* genomes and found 13 BphP homologs in 12 *Deinococcus* species (Figure 7A). The sequence identity between DrBphP and the other BphPs ranged from 35% (BphP of *Deinococcus* sp. UR1) to 64% (BphP of *Deinococcus* sp. YIM77859; Figure 7A). The same genetic structure (*bphO*-*bphP*-*bphR*) was observed in nine of the 13 *bph* operons reported here (Figure 7B). *Deinococcus wulumuqiensis*, which is the closest relative of *D. radiodurans* (Hong et al., 2015), does not encode any *bph* genes (Figure 7A). To test if the light-dependent increase in survival under chronic MMC exposure is conserved among the *Deinococcus* species, we tested representative species that contain *bphP*, *D*. *maricopensis*, or lack *bphP*, *D. wulumuqiensis*, and *D. proteolyticus* (Figure 7). *Deinococcus maricopensis* showed a more than 1-log increase in survival in the light, compared to the dark (Figure 8), similar to *D. radiodurans*. In contrast, no difference in survival was observed for *D. wulumuqiensis* and *D*. *proteolyticus* (Figure 8). These results show that the protective function of BphP upon chronic exposure to MMC in the light is conserved among the *Deinococcus* species.

## Discussion

In this study, we found that DrBphP playa protective role against chronic MMC exposure in the presence of light (Figure 2). The light-dependent protective role is conserved among several *Deinococcus* spp. possessing DrBphP homologs (Figures 7, 8).

The photoconversion of BphPs between Pr and Pfr differentially affects the biological activity of BphPs. DrBphP acts as a phosphatase, with higher activity in the Pfr state than in the Pr state (Multamäki et al., 2020). In this study, however, DrBphP enhanced MMC resistance in cells grown under both red and far-red light but not affect in those grown in the dark (Figure 2). The contradiction between the enzyme activity *in vitro* and physiological function *in vivo* is also observed in PsBphP1, one of the two BphPs encoded in the non-photosynthetic plant pathogen *Pseudomonas syringae*. PsBphP1 exhibits approximately 20-fold more efficient autophosphorylation in the Pfr form (Bhoo et al., 2001). However, PsBphP1 suppresses the swarming motility of *P*. *syringae* under both red and far-red light conditions and does not exert its effect in the dark (Wu et al., 2013). The conversion of Pfr to the Pr state can occur either by light absorption or by a light-independent thermal relaxation process, referred to as thermal or dark reversion. Although thermal reversion has been demonstrated *in vitro* for several phytochromes from different microbial species, its impacts on phytochrome signaling and physiological responses in these organisms remains unknown (Klose et al., 2020) and warrants further investigation.

Bacterial phytochromes act *via* various downstream partners. The purple nonsulfur bacterium *Rhodopseudomonas palustris* encodes six BphPs (RpBphP1–RpBphP6) of which only RpBphP2 and RpBphP3 display the typical BphP architecture (Auldridge and Forest, 2011). RpBphP2 and RpBphP3 and three RRs encoded in the same operon sense light quality and upregulate the synthesis of the photosynthetic apparatus, light-harvesting complex 4 (Fixen et al., 2019). In this study, two RRs, DrBphR and DR_0781, were found to be associated with DrBphP-dependent MMC resistance (Figures 5, 6). DrBphR had a negative effect on survival in the light during chronic MMC exposure (Figure 5). Since DrBphR is a substrate for the phosphatase DrBphP (Multamäki et al., 2020), it is likely that the light-activated DrBphP dephosphorylates DrBphR; this counteracts the negative effect of DrBphR, thereby protecting cells against MMC. In a typical TCS, the genes encoding HK and its cognate RR are organized in pairs. However, there also exist unpaired HKs and orphan RRs, i.e., an RR gene is not adjacent to a gene encoding HK, and vice versa. Despite obvious linearity in TCSs, functional coupling can occur between an orphan RR and a noncognate HK protein (Wang et al., 2009; Agrawal et al., 2016). Considering that DR_0781 is an orphan and also has a negative effect on cell survival similar to DrBphR (Figure 6), it is possible that DR_0781 is another substrate for DrBphP. It is worth investigating whether DR_0781 is dephosphorylated by DrBphP *in vitro*.

DrBphR is a single-domain RR comprising a receiver domain with no obvious regulatory domain, whereas DR_0781 is a two-domain RR containing a DNA-binding output domain (Galperin, 2010; Baker et al., 2016). Therefore, although how these RRs attenuate cell survival remains unclear, it can be inferred that the mechanisms underlying the negative effects of DrBphR and DR_0781 on survival are different. Notably, the genes encoding DNA repair proteins RecA, UvrA1, and UvsE were downregulated in Δ*bphR* and not in Δ*dr*_*0781*, and *recA* expression was enhanced by red light illumination in Δ*dr*_*0781* (Figure 6B). However, both Δ*bphR* and Δ*dr_0781* exhibited increased survival compared with WT, and a further increase in survival was observed in the *dr*_*0781 bphR* double mutant strain compared with Δ*bphR* or Δ*dr*_*0781* (Figure 6A). These results suggest that DrBphR and DR_0781 work independently of each other and affect different target genes or proteins, including DNA repair proteins, in response to light. Our future work will focus on identifying and investigating the roles of DNA repair proteins participating in the DrBphP-mediated increase in MMC resistance and their regulatory pathways.

## Data Availability Statement

The original contributions presented in the study are included in the article/Supplementary Material; further inquiries can be directed to the corresponding author.

## Author Contributions

J-HJ performed the experiments and wrote a first draft. SJ validated the experimental results. SI and M-KK constructed the mutants and plasmids. HS guided the experiments and interpreted the results. SL conceived the study, revised the first draft, and was in charge of overall direction and planning. All authors contributed to the article and approved the submitted version.

## Conflict of Interest

The authors declare that the research was conducted in the absence of any commercial or financial relationships that could be construed as a potential conflict of interest.

## Publisher’s Note

All claims expressed in this article are solely those of the authors and do not necessarily represent those of their affiliated organizations, or those of the publisher, the editors and the reviewers. Any product that may be evaluated in this article, or claim that may be made by its manufacturer, is not guaranteed or endorsed by the publisher.
