# Supplementary material for: Lack of the Bacterial Phytochrome Protein Decreases Deinococcus radiodurans Resistance to Mitomycin C
Source: Front Microbiol. 2021 Jul 30;12:659233. doi: 10.3389/fmicb.2021.659233 (PMC8363230; doi:10.3389/fmicb.2021.659233)
Supplement: Supplementary file 1 [file Data_Sheet_1.pdf]

Supplementary Table S1. Primers used in this study

Primer	Sequence (5' to 3')	
	forward*	reverse*
Mutant construction		
bphP-Up	gctggacagccaaaaagagc	cgctcaggcatcggtcgcgatcccggctcatgcg
bphP-Dn	cgcatgagccgggatcgcgaccgatgcctgagcg	gaggaaagcgggattcctgc
bphR-Up	agtggcggcagtacggcttc	cttttactgcggctcgcgacgctcaggcatcgg
bphR-Dn	ccgatgcctgagcgtcgcgagccgcagtaaaag	gtcagaggacgcgtttcggc
bphPR	tgatgcacttgcaagctgagc	agcgctttacgtgcttgaag
dr0408-Up	gtatctcgagaaggtatcagagcgcaaaa	tatgatatcggaaaaacctcgggaaagttcg
dr0408-Dn	tattctagactccgcctctcagctttcca	tatctgcagtgagttcggggatgcgcaa
dr0781-Up	gatactcgagccatcgccatcggaattc	gacagatatcgcgatgcccttcaaagatcg
dr0781-Dn	gatatctagaagcgcacctgaggaaagg	gatactgcagccaatcaaggcccacaaacc
dr0781-Up-Cam	attctcgagagcggctgcacttcaggg	gtatgatatctccggattgtcttcgatcaggag
dr0781-Dn-Cam	aatggatccgtggtggatgtccacatggc	tatctgcagagcgctccaggcagacact
dr0891-Up	gatactcgagccaccgctgcaaaccttg	gacagatatcggcaaaacctccggcaaaac
dr0891-Dn	gcgctctagagaaaatttaaccctctgcaacg	gatactgcagcaccgtgtacaccgtcaag
dr0987-Up	gtatctcgagctcgatcaagaacgtga	gtatgatatcagaaaaaccaccctgcgc
dr0987-Dn	gcgttctagaagcagttgacagaaaaagac	atatctgcagaagctgcgcgccatcga
dr1558-Up	gtatctcgaggacagcgaactaggagc	gtgagatatctgcccgtcttctttcagtac
dr1558-Dn	gtattctagaagagcgcaggcagcgac	gtatctgcagcagctccccggtttct
dr2556-Up	tatactcgagggaagtagcggtagtcg	gccagatatcatgagaagtctaacagttcg
dr2556-Dn	ggcatctagagggaagagaaaaaagaaacg	tatactgcaggtacacgtccgtccagg
drA0010-Up	gtatctcgaggctgttctggggaacc	gcatgatatcgacgttttctccagcagc
drA0010-Dn	gtattctagaagtgtggactggggctc	gtatctgcagctggtgtttctgatgg
drA0350-Up	ttggtaccatcaccttcgacgtgcccg	gtatgatatccccgctgtctcaagggacc
drA0350-Dn	gattctagatcgtgtcgagcaagagcgc	tatctgcagcaccaccggcacgcccag
drB0028-Up	ttggtaccagttcggtgccggaagtgg	gtatgatatcctggttgcccagccccag
drB0028-Dn	gattctagatggaaaccctgggggcca	tatctgcagctcgggggtctcatcggca
drB0029-Up	ttggtacccgcaacttcatcgccaacgc	gtatgatatcagcagcattgtcgtccacca
drB0029-Dn	gattctagatgtacaggacaacggtgtggg	tatctgcagaaccttcaacgctccgacct
drB0081-Up	gatactcgagaaatcggcgcggtggaca	tttagatatcggggcgcctcgggcagg
drB0081-Dn	gcgctctagatggccttttgttgtggatgg	gatactgcaggcagttcagcgctttcgag
Plasmid construction		
pBphP	tatgggcccatgagccgggacccgttgcc	tataagctttcaggcatcggcggctcccg
pBphR	tatgggcccatgcctgagcgcgcctctgt	gtataagcttttactgcggctggtacgtc

BphP-C24A	cggctcgcgctcggcgttctcggtggta	taccaccgagaacgccgagcgcgagccg
BphP-H532A	ggctcctgcatgtgggcgctgataacgaagcc	ggcttcgttatcagcgcccacatgcaggagcc
qRT-PCR		
pprI	cgaagcgttccagcatt	gcggctcgaacaggtcat
recA	cgattgatgtggtggtcgt	acctggttgatgaagatggc
uvrA1	cgctgcactacgagtacgac	cacgtccaccagaaacttca
uvsE	ccaagctgctcgacctgtat	ggctcgaactcagtcggtag
gap	gtgaacgacgagcagtacga	gtggtcatgatggccttctc

* Restriction enzyme sites are underlined.

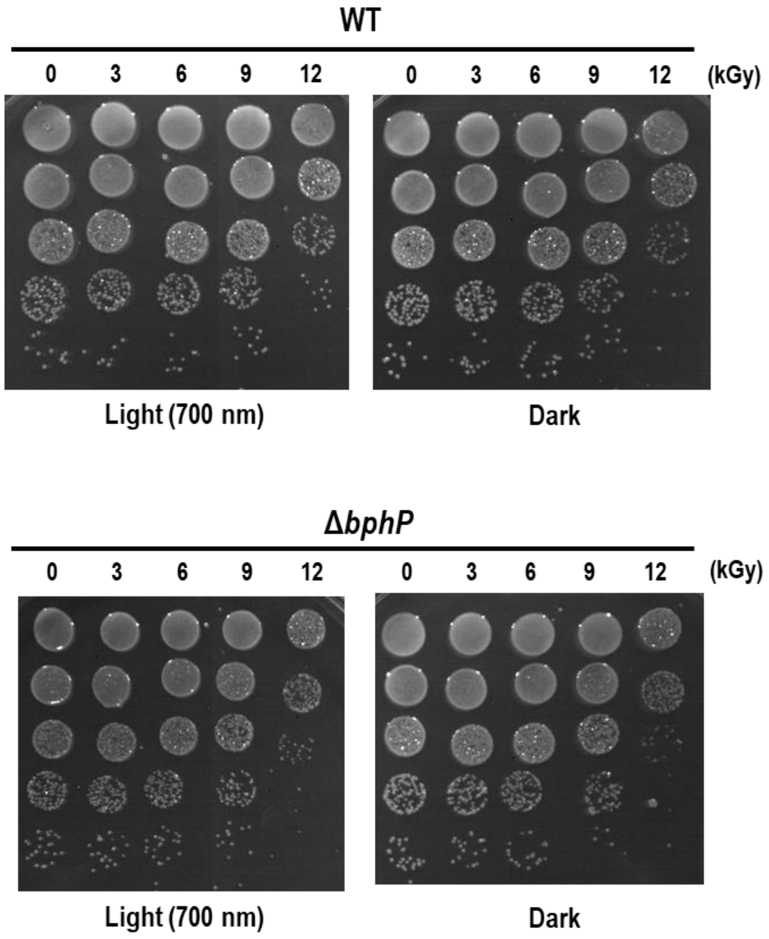

Supplementary Figure S1. Survival of Δ*bphP* in response to acute γ-radiation exposure. *D. radiodurans* wild-type (WT) and *bphP* mutant (Δ*bphP*) strains were grown to log phase, treated with γ-radiation at a dose rate of 6 kGy/h, and then plated on TGY. The plates were exposed UVC radiation at the indicated doses and then incubated under light (red light, 700 nm) and no light (dark) conditions for 2 days.

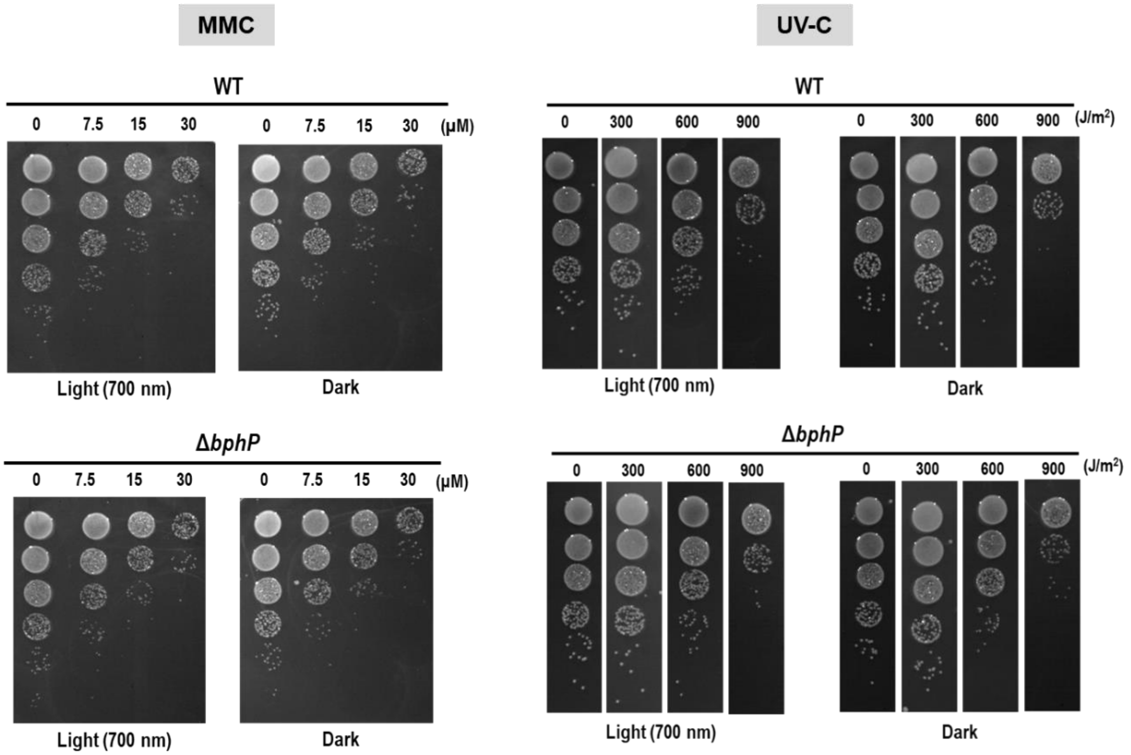

Supplementary Figure S2. Survival of Δ*bphP* in response to acute MMC and UV-C exposure. *D. radiodurans* wild-type (WT) and *bphP* mutant strains were grown to log phase, treated with MMC for 1 h at the indicated concentrations, and then plated on TGY. For UV-C exposure, cells first spotted on TGY plates, and the plates were exposed UV-C radiation at the indicated doses. The plates were incubated under light (red light, 700 nm) or dark (no light) conditions for 2 days.

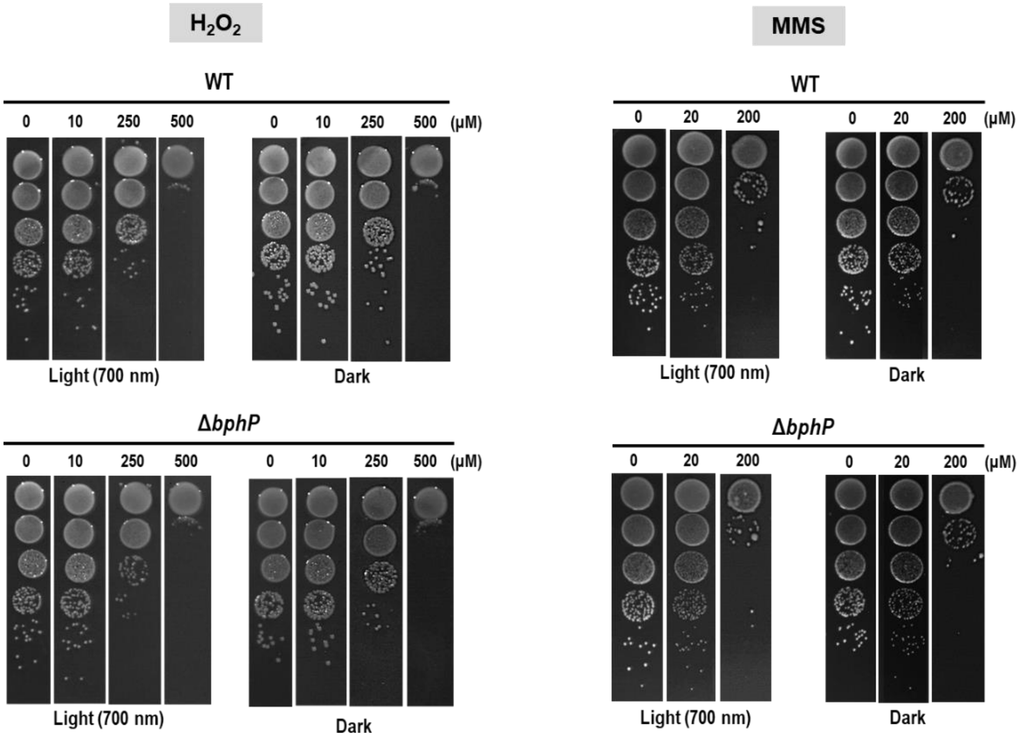

Supplementary Figure S3. Survival of Δ*bphP* during continuous exposure to H_2O_2 and MMS. *D. radiodurans* wild-type (WT) and *bphP* mutant strains were grown to log phase, then serially diluted, and spotted on TGY plates supplemented with the indicated concentrations of H_2O_2 and MMS. The plates were incubated under light (red light, 700 nm) or no light (dark) conditions for 2 days.

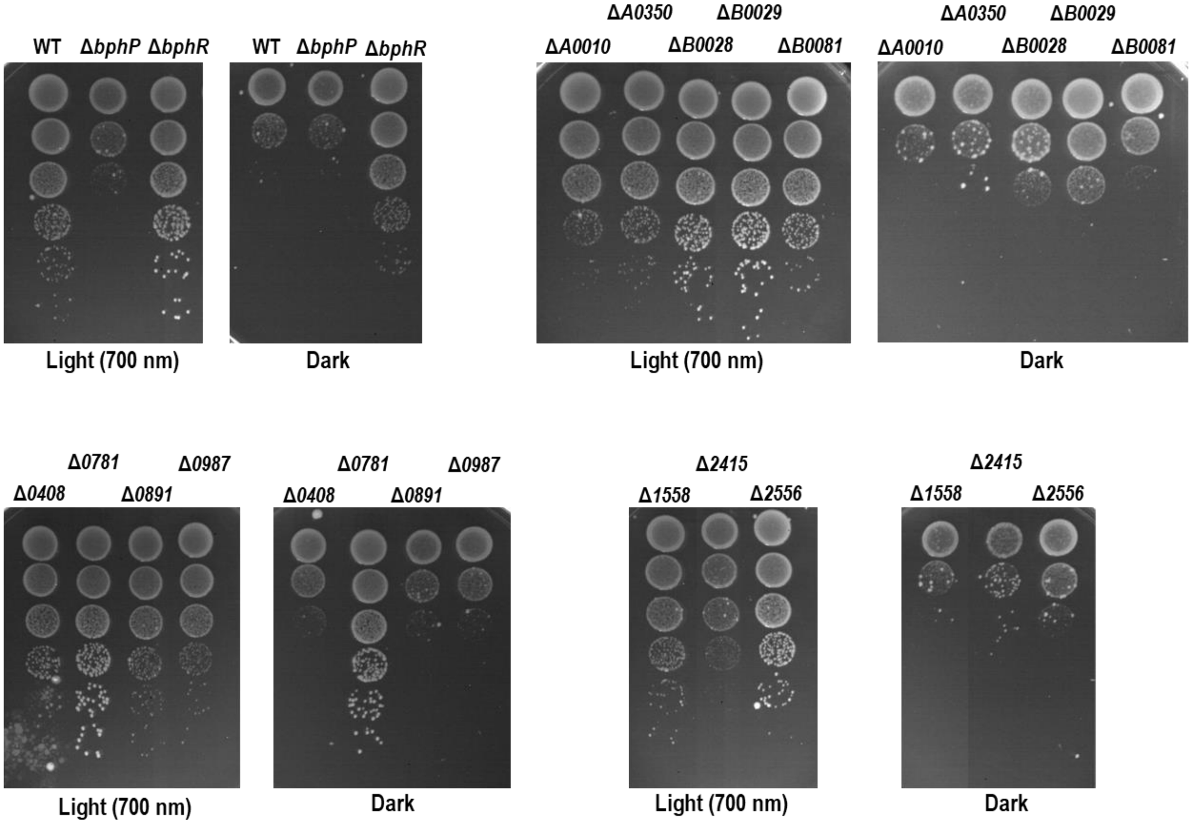

Supplementary Figure S4. Survival of *D. radiodurans* RR mutant strains. Each of the indicated mutant strains were grown to log phase and spotted on TGY plates supplemented with 300 nM MMC. The plates were incubated under light (red light, 700 nm) or no light (dark) conditions for 2 days.

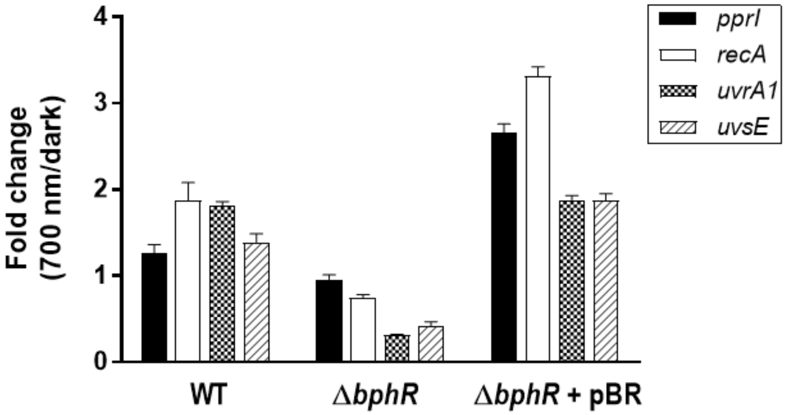

Supplementary Figure S5. qRT-PCR assay. Cells were grown to log phase (OD_{600} ≈ 1.0) in the presence of MMC (300 nM) under red light (700 nm) and dark conditions, respectively. The mRNA levels were measured in WT, Δ*bphR*, and Δ*bphR* with the plasmid pBR encoding DrBphR. The relative expression values of indicated genes were determined by dividing the mRNA levels from the illuminated cells by the mRNA levels from cells kept in the dark. The expression levels of the target genes were normalized against *dr_1343*. The data is represented as the mean ± standard deviation of three independent experiments performed in duplicate.
